# Extremophilic Microorganisms for the Treatment of Toxic Pollutants in the Environment

**DOI:** 10.3390/molecules25214916

**Published:** 2020-10-23

**Authors:** Sun-Wook Jeong, Yong Jun Choi

**Affiliations:** School of Environmental Engineering, University of Seoul, Seoul 02504, Korea; jeongsunwook@gmail.com

**Keywords:** bioremediation, toxic pollutants, extreme conditions, extremophilic microorganism

## Abstract

As concerns about the substantial effect of various hazardous toxic pollutants on the environment and public health are increasing, the development of effective and sustainable treatment methods is urgently needed. In particular, the remediation of toxic components such as radioactive waste, toxic heavy metals, and other harmful substances under extreme conditions is quite difficult due to their restricted accessibility. Thus, novel treatment methods for the removal of toxic pollutants using extremophilic microorganisms that can thrive under extreme conditions have been investigated during the past several decades. In this review, recent trends in bioremediation using extremophilic microorganisms and related approaches to develop them are reviewed, with relevant examples and perspectives.

## 1. Introduction

**Figure 1:**
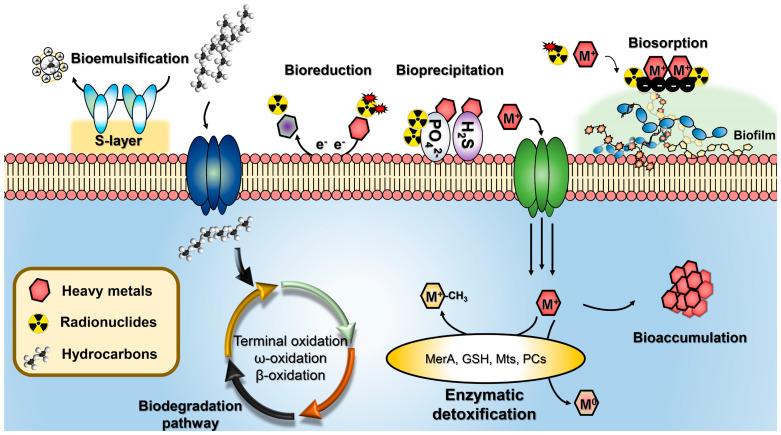
Current microbial bioremediation strategies for the removal of diverse toxic pollutants. Biosorption, a metabolically independent process based on ionic interactions between the extracellular surface of biomass and metal ions; bioaccumulation, a metabolically active process in which microorganisms use proteins to absorb metal ions inside their intracellular space; bioprecipitation, a process of immobilizing soluble metal ions through redox reactions, enzymes, and metabolites on the extracellular surface of microorganisms; bioreduction, a process of transformation of toxic metals/metalloids to non-toxic elements through a biological reduction and oxidation process; bioemulsification, a biological process of using proteins or metabolites to form emulsions in two immiscible liquid phases.

Due to the rapid industrial growth, the environment and public health are threatened by the huge amount of toxic pollutants that have accumulated in the environment. Therefore, maintaining and protecting the environment from toxic pollutants has become a great challenge for mankind over the past few decades. Recently, various strategies have been intensively exploited to protect the environment by preventing the dispersion of toxic pollutants into it. For example, physicochemical methods such as electrochemical treatments, excavation, ion exchange, precipitation, reverse osmosis, evaporation, and sorption have been developed for the removal of toxic substances [1,2,3,4]. However, many of these techniques are not yet commonly applied to the actual treatment of contamination due to critical drawbacks such as high cost and secondary contamination possibly associated with them [5,6,7]. As an alternative, microbial bioremediation has attracted much attention as a promising technology that can overcome the shortcomings of the currently used physicochemical methods (Figure 1) [8,9,10]. Specifically, extremophilic microorganisms offer the most suitable approach for the treatment of toxic pollutants [11,12,13,14] because not only can they detoxify toxic pollutants through microbial cellular metabolism but also they can withstand extremely harsh conditions [11,13,14,15]. Herein, we focus on recent trends in bioremediation processes for the treatment of toxic pollutants such as inorganic heavy metals, harmful organic substances, and radioactive elements using extremophilic microorganisms and on the perspectives of this approach in public health.

## 2. Survival Strategies of Extremophilic Microorganisms under Extreme Conditions

Extreme environments are defined as habitats that make the prospect of survival difficult for most organisms on earth. These are mostly natural conditions such as extreme temperatures, salinity, pH, and desiccation observed in environments such as deep sea, volcanoes, and deserts. However, these extreme conditions can also appear in polluted areas containing harmful organic substances [16], heavy metals [17], and/or radioactive waste [18]. Under extremely polluted conditions, the clean-up process of pollutants by using physicochemical methods is not always successful due to limited accessibility to the pollutants and secondary contamination. Thus, there is a need to combine microbial biotechnology and chemistry to advance the remediation processes. Over the past century, extremophilic microorganisms have adapted and evolved in various ways to thrive under extreme conditions through unique biological mechanisms. During the process of adaptation, extremophilic microorganisms have evolved not only to convert unstable toxic pollutants into sufficiently stable beneficial resources for their cellular metabolism but also to become highly tolerant to toxic matter. Thus, many studies have been attempted to develop sustainable bioremediation processes using the survival strategies of extremophilic microorganisms. Here, we briefly describe the adaptation and survival mechanisms that can be used for bioremediation.

### 2.1. Acidophilic and Alkaliphilic Microorganisms

Acidophilic microorganisms can survive under extremely low pH (less than pH 3) conditions, maintaining pH homeostasis by controlling proton permeation [19]. For example, microorganisms from the genera *Thermoplasma*, *Ferroplasma,* and *Sulfolobus* can regulate proton permeation under extremely low pH conditions due to a highly impermeable cell membrane mainly composed of tetraether lipids having a diverse array of polar head groups and a bulky isoprenoid core [20,21,22,23]. The modulation of the influx of protons through the proton pump system is important to survive at low pH, and putative proton pump proteins such as H^+^-ATPase, symporters, and antiporters from *Ferroplasma* type II and *Leptospirillium* group II are involved in maintaining pH homeostasis [21,24,25]. Moreover, F_0_F_1_-type adenosine triphosphate synthase in *Bacillus acidocaldarus*, *Thermoplasma acidophilum*, and *Leptospirillium ferriphilum* is known to play a critical role in regulating proton permeation [25]. In addition to these mechanisms, several other auxiliary mechanisms, involving for example, chaperone proteins and cytoplasmic buffering capacity contribute to survival strategies under extremely low pH conditions by protecting intracellular molecules such as DNA, RNA, and proteins [25].

Contrary to acidophilic microorganisms, alkaliphilic microorganisms can resistant high pH. To date, three key biological mechanisms have been identified as survival strategies in these microorganisms. First, under extremely high pH conditions, some alkaliphilic *Bacillus* spp. can increase the generation of proton motive force through synthesizing a secondary acidic cell membrane consisting primarily of peptidoglycan, teichuronic acid, and teichuronopeptide [26,27]. Increasing the proton motive force contributes to not only energy generation but also pH balance [28,29,30]. Second, sodium motive force can also promote pH balance under extremely high pH conditions [31,32]. Under high Na^+^ ion conditions, Na^+^/H^+^ antiporters extrude Na^+^ ions and absorb a greater amount of extracellular H^+^ ions than that of extruded Na^+^ ions, thereby activating a bioenergetic process and regulating the internal pH [33]. Finally, the production of organic acids that can be used for pH calibration is known to be an important biological process in maintaining pH balance [34,35].

### 2.2. Halophilic Microorganisms

Halophilic microorganisms can thrive in a high-salt environment which hinders organisms’ survival due to osmolar imbalance and metabolic problems [36,37]. Previous studies on halophilic microorganisms reported two fundamental adaptation strategies to survive under extremely high salt conditions. The first is to use a “salt-in” strategy that refers to the accumulation of inorganic osmoprotectants such as KCl inside the cell to maintain the osmotic balance both inside and outside the cell [37]. It has been demonstrated that *Halobacterium salinarum* can accumulate 3.97 M and 4.57 M of K^+^ and Cl^−^ ions, respectively, inside the cell using the ATP-dependent K^+^ transport system (the KdpFABC complex and cationic amino acid transporter-3 (Cat3) and Na^+^ efflux antiporters (NhaC) to balance the osmotic gradient under high-salt conditions [38,39,40,41]. Moreover, halophilic microorganisms have evolved an abundance of negatively charged aspartate and glutamate residues on protein surfaces that can interact with water molecules to form a water cage that prevents protein precipitation and dehydration [41,42,43,44].

As another adaptation strategy, some halophilic and halotolerant bacteria use the ‘compatible solutes adaptation’ strategy to maintain osmotic balance by using compatible organic solutes such as polyols, glucosylglycerol, sucrose, trehalose, ectoine, and betaine [45,46]. For example, the halophilic bacterium *Spiribacter salinus* M19-40 produces enhanced levels of compatible solutes such as ectoine and trehalose when they are exposed to a high NaCl concentration [45]. These organic solutes have a critical role in reducing the thermodynamic activity of water to compensate for the external osmotic pressure [47].

### 2.3. Psychrophilic and Thermophilic Microorganisms

Psychrophilic microorganisms usually have a preferred temperature range of 1–4 °C. Unlike mesophilic microorganisms, whose preferred temperature range is 30–37 °C, psychrophilic microorganisms can fully maintain cellular metabolism even at temperatures below 0 °C. To adapt to these harsh conditions, they have evolved several physiological adaptation mechanisms, including membrane fluidity control, molecular chaperones’ action, and antifreeze molecules’ synthesis [48,49]. For example, they can modulate membrane fluidity by altering its lipid composition, increasing the amount of polyunsaturated fatty acids and polar/non-polar carotenoids and decreasing the size of the lipid head groups [19,49]. A variety of temperature-induced enzymes such as cold-shock proteins (Csps) and heat-shock proteins (Hsps) are also involved in cold-shock resistance by regulating signaling cascades that protect damaged proteins and cofactors [50]. Moreover, various antifreeze proteins and polysaccharides such as trehalose, mannitol, and exopolysaccharides, which are constituents of biofilm, can act as cryoprotectants [51].

Thermophilic microorganisms with a preferred temperature above 60 °C activate similar survival mechanisms to psychrophilic microorganisms. For example, *B. acidocalidus*, a thermophilic spore-forming bacterium, modulates membrane lipid fluidity by increasing hopanoids (a subclass of triterpenoids) to resist high temperatures [52]. The thermophilic archaeon *Metahnocaldococcus jannaschii* can resist high temperatures by regulating membrane lipid composition. When these microorganisms were exposed to high temperature, the diether lipids decreased from 80% to 20%, while the caldarchaeol-based and cyclic archaeol-based lipids increased from 10% to 40% [53,54]. In addition, thermophilic microorganisms have evolved various biomolecules to induce thermal stability, e.g., by increasing the guanine/cytosine content of DNA or developing a positive supercoiled DNA structure [55]. Moreover, they not only possess very rich ribosomal proteins but also have a well-developed heat-shock response to allow normal protein synthesis even at high temperatures [56,57].

### 2.4. Radiophilic Microorganisms

Radiophilic (radio-tolerant) microorganisms can thrive in environments with high levels of radiation, including ultraviolet light and gamma rays. Previous studies on how they can adapt and survive under high-dose radiation and oxidative stress conditions have revealed that they possess robust DNA repair systems and antioxidation mechanisms to withstand intensive irradiation stress [58,59,60,61,62,63]. For example, RecA proteins from *Deinococcus radiodurans* R1, which is a representative radiophilic microorganism, plays a crucial role in repairing damaged DNA under gamma ray irradiation [63,64]. When it is exposed to a high dose of irradiation, the expression levels of several novel proteins (PprA, PprM, PprI, and DdrABCDO) and of DNA damage response regulons are dramatically increased and contribute to DNA repair and damaged genome reconstruction [65,66,67,68].

Radiophilic microorganisms also have efficient antioxidant enzymes, such as catalase (CAT), superoxide dismutase (SOD), and peroxidase, which are responsible for the scavenging of reactive oxygen species (ROS) [63,69]. For example, CATs and SODs from *D. radiodurans* exhibit a 30-fold higher ROS scavenging activity than radiation-sensitive bacteria such as *Escherichia coli* and *Saccharomyces cerevisiae* [63]. Moreover, non-enzymatic factors such as relatively high intracellular manganese concentrations, polyphosphate granules, carotenoids, and pyrroloquinoline quinone are also involved in the efficient scavenging of various ROSs as well as in the protection against protein damage [70,71,72,73]. Other non-enzymatic factors protecting biomolecules from ionizing radiation are a high intracellular Mn/Fe concentration ratio, orthophosphates, large amounts of free amino acids, and small peptides that have been found in the polyextremophilic microorganism *H. salinarum* [74].

## 3. Bioremediation Using Extremophiles

### 3.1. Treatment of Heavy Metal Pollutants

Concerns about the toxicity of heavy metals have been drastically increasing because even a tiny amount can be dangerous for public health and the environment. Moreover, currently used chemical treatments of toxic heavy metals under extreme conditions is often hampered by their poor accessibility. Thus, the development of sustainable bioremediation methods using extremophilic microorganisms for the treatment of heavy metals has been investigated during the past several decades (Table 1). In the case of extremely acidic conditions, acidophilic microorganisms that can thrive under low pH conditions have been used as host strains for the detoxification of heavy metals through biomining processes such as bioleaching and bio-oxidation [75,76,77,78]. There have been several reports on the development of bioremediation processes using *Acidothiobacillus* strains, which are the most common acidophilic and chemolithotrophic microorganisms. For example, industrial-scale bioleaching has been performed using *Acidothiobacillus ferrooxidans* [79,80,81]. Romero-González et al. [82] reported the bioremediation of 100 mg/L of U(IV) ex situ from polluted mine water using *At. ferrooxidans* NCIMB 8455, while Jameson et al. [83] demonstrated the utility of *At. ferrooxidans* and *Acidothiobacillus ferrivorans* strains for hydrogen sulfide (H_2_S)-assisted copper precipitation (>99%) under acidic conditions (pH 2.5–2.6). In other studies, the efficient reduction of vanadium ions [vanadate; V(V)] to V(IV) and the biosorption of cadmium cations were successfully achieved by *Acidocella aromatica* PFBC and *Acidiphilium symbioticum* H8, respectively, under highly acidic conditions [84,85].

More efficient decontamination of toxic heavy metals can be obtained using a microbial consortium, a major advantage of which is to synergize different enzymatic systems and metabolic pathways of individual microorganisms. Recently, the bioaugmentation of heavy metals using an acid mine drainage (AMD)-isolated acidophilic microorganism consortium was performed on polluted port sediment. The extraction of more than 90% Cu^2+^, Cd^2+^, Hg^2+^, and Zn^2+^ was successfully achieved using an acidophilic microbial consortium consisting of *Acidothiobacillus thiooxidans, At. ferrooxidans, Acidiphilium cryptum,* and *Leptospirillum ferrooxidans* [86]. Another study also reported the in situ bioremediation of AMD soil defined as highly acidic (pH 3.21), sulfate (6285 mg/L), and heavy metals. The introduction of an enriched microbial consortium composed of acidophilic microorganisms and metal-resistant strains of *Chloroflexi* (29%), *Acidobacteria* (21%), *Proteobacteria* (16%), and *Firmicutes* (2%) into AMD soil enabled 97% reduction of dissolved sulfate and increased the pH to 7.5 [87].

Halophilic microorganisms offer great advantages in the treatment of toxic pollutants in high-salt environments. For example, bioremediation using marine bacteria is a promising solution for the decontamination of seawater from toxic heavy metals, as these bacteria can survive at high salt concentrations. There have been a few reports on the removal of toxic heavy metals using several marine bacteria. For instance, *Vibrio harveyi* showed a good capability to accumulate cadmium cations inside the cell with a high adsorption capacity (up to 23.3 mg Cd^2+^/g of dry cells) [88]. Another marine bacterium, *Enterobacter cloaceae*, can chelate Cd, Cu, and Co by up to 65%, 20%, and 8%, respectively, from mixed-salts solutions [89]. In addition to marine bacteria, some thermophilic microorganisms such as *Geobacillus thermantarcticus* and *Anoxybacillus amylolyticus* have considerable biosorption capacity for heavy metals, which suggests their applicability for the removal of heavy metals in polluted environments [90].

As the development of biotechnology progresses, more advanced bioremediation methods that are superior to traditional methods have been reported. Unlike conventional bioremediation methods whose principle is based on the microorganism itself, new methods present improved efficiency and specificity thanks to the use of biomolecular engineering approaches. For instance, S-layer proteins, which have high stability and activity toward various heavy metals, are produced by lactic acid bacteria and are promising biomolecules for toxic heavy metal decontamination under very low pH (pH 2) conditions [91]. The S-layer proteins from *Lactobacillus plantarum* YW11 showed 99.9% Pb adsorption capacity [92]; scanning electron microscopy–energy dispersive X-ray analysis demonstrated that the Pb^2+^ ions were efficiently adsorbed and accumulated on the cell surface of *L. plantarum* YW11 in a process mediated via S-layer proteins. The interaction of S-layer proteins from two *Lactobacillus kefiri* strains (CIDCA 8348 and JCM 5818) has also been investigated for the adsorption of various metal ions such as Cd^2+^, Zn^2+^, Pb^2+^, and Ni^2+^ [93].

### 3.2. Biodegradation of Organic Pollutants

A variety of microorganisms can transform toxic organic pollutants into non-toxic substances such as petroleum hydrocarbons, aromatic petrochemicals, and various halogenated compounds (Table 2). Such complete transformation requires not only strong resistance to toxic organic pollutant exposure but also the ability to utilize toxic organic contaminants for their cellular metabolism. Therefore, extremophilic microorganisms that have adapted to harsh environments such as extreme temperatures and high salt concentrations over a long time period can potentially be widely used for the treatment of organic toxic pollutants under the corresponding condition. For example, the decontamination of polycyclic aromatic hydrocarbons and long-chain alkanes (C_10_ to C_32_) using thermophilic *Bacillus*, *Thermus*, and *Geobacillus* strains isolated from oil-contaminated areas has been reported [95,96,97,98,99]; a *Geobacillus* SH-1 strain isolated from a deep oil well was also able to degrade saturated alkanes ranging from C_12_ to C_33_ and naphthalene. In another study, C_12_–C_21_ *n*-alkanes were completely decomposed within 8 days, and 100 ppm of naphthalene was almost degraded within 72 h [100]. Furthermore, bioaugmentation through introduction of various extremophilic microorganisms including *Geobacillus thermoparaffinivorans* IR2, *Geobacillus stearothermophilus* IR4, and *Bacillus licheniformis* increased the decontamination of long alkyl (C_32_ and C_40_) substances [101].

In addition to thermophilic microorganisms, psychrotrophic and halophilic microorganisms have shown excellent performance in the treatment of organic hydrocarbon pollutants. Low-temperature-adapted *Pseudoalteromonas* sp. P29 and *Oleispira antarctica* RB-8^T^ exhibited high efficiencies in the degradation of hydrocarbon mixtures composed of diesel, military jet fuel, and crude oil [102,103], while the halotolerant microorganisms *Marinobacter sedimentalis*, *Marinobacter falvimaris*, and *Marinobacter nanhaiticus* D15-8W were able to transform biphenyl, phenanthrene, anthracene, and naphthalene into useful carbon sources in hypersaline environments (e.g., salt lakes, salt marshes, and highly saline soils) [104,105]. In particular, extracellular polymeric substances (EPSs), which are cellular components of halophilic microorganisms, play a critical role in the remediation of organic pollutants from hypersaline environments. Exopolysaccharides secreted by halophiles can act as biosurfactants that contribute toward aggregating oils and emulsifying hydrocarbons, as well as offer cellular resistance toward toxic heavy metals. Halophilic microorganism *Halobacillus* sp. EG1HP4QL develops the ability to utilize crude oil as the sole carbon source within 12 days and to degrade paraffin (34.5%), naphthalene (49.6%), mono- and bicyclic aromatic hydrocarbons (51.2%), polycyclic aromatic hydrocarbon (43.5%), and alcohol–benzene resins (25.5%) [106]. EPS-producing *Halomonas* strain TG39 was also used for bioremediation of a hydrocarbon-contaminated Deepwater Horizon spill site [107]; the extracted EPS was effective not only in increasing the solubilization of aromatic hydrocarbons but also in enhancing the degradation rate of phenanthrene. Hence, bioremediation using extremophilic microorganisms is a promising method for the treatment of organic contaminant-polluted areas under extreme conditions because the organic pollutants can be metabolized by the microorganisms.

### 3.3. Microbial Treatment of Radioactive Waste

Recent advances in synthetic chemistry and separation methods have led to the design of various adsorbent systems including surface-modified nanomaterials and/or hybrid composites for the treatment of radionuclides in soil or aqueous media. For example, surface-modified iron oxide (Fe_3_O_4_) nanoparticles have been applied to selectively adsorb toxic heavy metals such as Cr(III), Co(II), Ni(II), Cd(II), Pb(II), and As^3+^ from aqueous media [108]. Furthermore, engineered Au nanomaterials have been developed that are excellent adsorbents for the desalination of non-radioactive and radioactive iodine anions [109,110,111]. However, there are still several problems in the practical application of these methods. First, a large volume of secondary radioelement-contaminated solid adsorbents is generated during the desalination procedure, and so the removal of unsettled adsorbents after the treatment requires an additional expensive step. Second, small- (nano- or micro-) sized adsorbents tend to lose their stability and properties under particularly harsh conditions such as high salt concentration and high radiation. Therefore, employing extremophilic microorganisms that can be used as a live cleaning agent offer a useful alternative for the treatment of radioactive waste (Table 3).

The microbial treatment of radioactive waste can be accomplished through the interactions between microorganisms and radioisotopes, such as biomineralization, biotransformation, and biosorption [112,113,114,115]. Among these, mineralization of the target element inside bacterial cells has been proposed as the main strategy for the removal of radionuclides from a contaminated area [116,117]. As an example, *Shewanella* and *Geobacter* strains can reduce some alpha nuclides such as U(VI), Pu(IV), Am(V), and Th(IV) to make them harmless [15,114,116,118,119]. Anderson et al. reported the removal of uranium from aqueous media by using acetate-stimulating *Geobacter* species, while enhanced removal efficiency was demonstrated by supplementation with glucose, ethanol, and acetate as an electron donor [120]. Since the 1990s, a variety of extremophilic microorganisms that can thrive under high levels of ionizing radiation conditions (>15 kGy) have been identified [121,122,123]. Among these, *D. radiodurans,* which is one of the most radio-resistant microorganisms, has received much attention as a biological material for on-site treatment of radionuclide-contaminated environments [124,125] (Table 3). Moreover, a variety of studies investigating the development of the bioremediation processes using *D. radiodurans* for the removal of radionuclides pollutants have been reported [123,126,127,128,129]. A genetically engineered *D. radiodurans* strain expressing a non-specific acid phosphatase from *Salmonella enterica* serovar Typhi [127,128,129] or bacterial Ni/Co transporter (NiCoT) [130] can precipitate the oxidized form of uranium pollutants and radioactive cobalt (^60^Co), respectively.

In recent years, the combination of extremophilic microorganisms with nanotechnology has emerged as a central strategy in efforts to treat polluted environments. A few case studies including the biosynthesis of various nanomaterials using extremophilic microorganisms have been reported [131,132,133,134,135]. With the advent of nano-biotechnology, the combination of extremophilic microorganisms with nanomaterials (nano-adsorbents and reductants) will be a promising technology for useful bioremediation applications. For example, a highly efficient and stable method for the removal of radioactive iodine (^125^I) using *D. radiodurans* with biogenic Au nanoparticles has been reported [131], in which more than 3.7 MBq of ^125^I was efficiently removed (>99%) within 30 min. More recently, the thermo-acidophilic archeon *S. tokodaii* 7^T^ (NBRC 100140) capable of synthesizing biogenic Pd(0) nanoparticles (mean diameter: 8.7 nm) showed four-fold increased Cr(IV) reduction with 2.0 mg Cr(VI)/L/h/Pd(0) compared to a commercial Pd/C catalyst [(0.5 mg Cr(VI)/L/h/Pd(0)] [136]. Another study also demonstrated efficient Cr(IV) reduction using Pd(0) nanoparticles synthesized by the acidophilic Fe^3+^-reducing bacteria *Ac. aromatica* PFBCT and *Ap. cryptum* SJH via a one-step microbiological reaction [137].

## 4. The Future Direction

Pollution, which has emerged as a side effect of the rapid growth of industrialization and urbanization, is a worldwide threat to the environment and public health. Thus, the development of highly efficient and stable methods for cleaning up polluted environments has become a major challenge. Although a variety of conventional methods to remove toxic pollutants have been developed over the past several decades, there are still many hurdles that need to be overcome to realize practical applications [138]. Hence, extremophilic microorganisms, which can thrive under harsh conditions, have been receiving particular interest as bioagents for the removal of toxic pollutants.

Although conventional microbial bioremediation processes have succeeded in the removal of various toxic pollutants, current methods still require much effort to overcome their limitations in terms of cost-effectiveness, removal efficiency, and practicality. *E. coli* and *Bacillus* spp. are commonly considered host strains for microbial bioremediation processes, being well known due to their broad use with well-established genetic engineering tools [139,140]. However, despite intensive genetic engineering, the practical use of these microorganisms for on-site remediation is extremely limited, owing to their relatively weak resistance to harsh conditions and low removal efficiency. Thus, to overcome these limitations, subsequent strategies based on the combination of extremophilic microorganisms with advanced biotechnology from fields such as systems metabolic engineering, synthetic biology, and nanotechnology have enhanced the performance of bioremediation through reprogramming the nature of wild-type microorganisms [141,142]. Several approaches based on biotechnology and nanotechnology are (**1**) screening and identification of microorganisms that have a strong tolerance for harsh conditions, (**2**) making microorganisms capable of degrading a variety of environmental toxic pollutants, (**3**) increasing the removal capacity and specificity of microorganisms toward target pollutants, and (**4**) expanding the removal spectrum of microorganisms using biogenic nanoparticles. Moreover, a variety of advanced tools in bioengineering, such as in silico flux analysis, biostatistics, and multi-omics analysis, will allow us to access the possibly infinite potential of extremophilic microorganisms for the treatment of environmental toxic pollutants.

## 5. Conclusions

**Figure 2:**
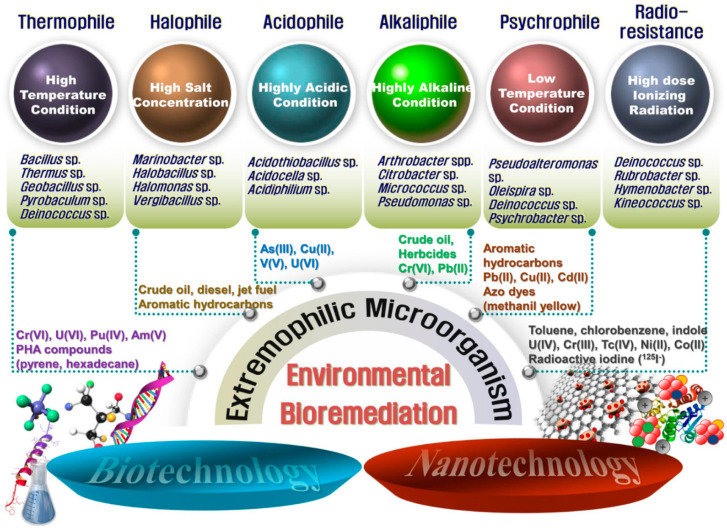
A schematic diagram of advanced bioremediation using extremophilic microorganisms combined with biotechnology and nanotechnology. Representative candidates that can be used as a host strain for the treatment of pollutants in the environment are shown.

When considering all the aspects presented in this review, extremophilic microorganisms appear as attractive bioagents for the clean-up of toxic pollutants contaminating the environment, due to their unique characteristics such as toughness, adaptability, and strong resistance to extreme conditions. Although many challenges still need to be addressed, the adoption of extremophilic microorganisms for the development of bioremediation processes is an environmental imperative for us to meet the needs of global public health. Indeed, combining extremophilic microorganisms with biotechnology and nanotechnology will open new avenues toward developing highly efficient and eco-friendly methods for the treatment of toxic pollutants (Figure 2).

## Figures and Tables

**Table 1:** Extremophilic microorganisms used in the removal of heavy metals.

Heavy Metal	Method/Mechanism	Extremophile	Resistance ^1^	Removal Efficiency (Initial Concentration) ^2^	Reference
As(III)	Bioleaching ^3^	*Acidothiobacillus ferrooxidans* BY-3	Low pH (pH < 1.8)	35.9% (ND)	[80]
U(VI)	Bioleaching	*At. ferrooxidans*	Low pH (pH 1.5–4.5)	50% (100 mg/L)	[82]
Cu(II)	Bioprecipitation	*Acidothiobacillus ferrivorans*	Low pH (pH 2.5)	>99% (50 mM)	[83]
V(V)	Bioreduction	*Acidocella aromatica*	Low pH (pH 2.5)	70% (1 mM)	[84]
Cd(II)	Biosorption	*Acidiphilium symbioticum* H8	ND	248.62 mg Cd(II)/g biomass (250 mg/L)	[85]
	Bioaccumulation	*Vibrio harveyi*	60 mg/L MIC	84% (30–60 mg/L)	[88]
	Biosorption	*Enterobacter cloaceae*	ND	65% (100 mg/L)	[89]
	Biosorption	*Geobacillus thermantarcticus, Anoxybacillus amylolyticus*	High temperature (80 °C)	85.4%, 74.1% (50 mg/L)	[90]
Cr(VI)	Bioreduction	*Pyrobaculum islandicum*	High temperature (100 °C)	100% (600 μM)	[94]

^1^ Either the experimental conditions or the tolerance of the species. ND, not determined; MIC, minimum inhibitory concentration. ^2^ Initial concentration of contaminant in the test. ^3^ Bioleaching, a metal solubilization process mediated by sulfur-/iron-oxidizing bacteria.

**Table 2:** Extremophilic microorganisms used in the removal of hydrocarbons.

Hydrocarbons	Extremophile	Resistance	Removal Efficiency (Initial Concentration)	Reference
acenaphthene, fluoranthene, pyrene, benzo[e]pyrene	*Bacillus* spp., *Thermus* sp.	High temperature (60–70 °C)	35–77% (30–60 mg/L)	[95]
Pentadecane, octadecane, octacosane	*Geobacillus* sp. SH-1	High temperature (70 °C)	>70% (100 mg/L)	[100]
Rotricontane, tetracotane	*Geobacillus thermoparaffinivorans,* *Geobacillus stearothermophillus, Bacillus licheniformis*	High temperature (50 °C)	>87% (1 g/L)	[101]
Mixed oil	*Pseudoalteromonas* sp. P29	Low temperature (5 °C)	90% (2 g/L)	[102]
Diesel, jet fuel, crude oil	*Oleispira antarctica* RB-8T	Low temperature (4–15 °C)	53.7–79.4% (1 g/L)	[103]
Biphenyl, phenanthrene, anthracene, naphthalene	*Marinobacter sedimentalis,* *Marinobacter falvimaris,* *Marinobacter nanhaiticus*	High salinity (5M NaCl)	70–90% (0.2–3 g/L)	[104,105]

**Table 3:** Extremophilic microorganisms used in radioactive waste bioremediation.

Radionuclide	Extremophile	Resistance	Removal Efficiency	Reference
U(VI), Cr(VI), Tc(VII)	*Deinococcus geothermalis*	Radiation (12 kGy), high temperature (55 °C)	>90%	[123]
	*D. radiodurans*	ND	95–100%	[126]
U(VI)	*D. radiodurans* expressing PhoN	Radiation (6 kGy)	>90%	[127]
Co-60	*D. radiodurans* expressing NiCoT	Radiation (6.4 kGy)	>60%	[130]
I-125	*D. radiodurans*	Radiation (8 kGy)	>99%	[131]

## References

[1] Muddemann T., Haupt D., Sievers M., Kunz U. (2019). Electrochemical reactors for wastewater treatment. ChemBioEng Rev..

[2] Ouyang W., Chen T., Shi Y., Tong L., Chen Y., Wang W., Yang J., Xue J. (2019). Physico-chemical process. Water Environ. Res..

[3] Xiang Q., Nomura Y., Fukahori S., Mizuno T., Tanaka H., Fujisawa T. (2019). Innovative treatment of organic contaminants in reverse osmosis concentrate from water reuse: A mini review. Curr. Pollut. Rep..

[4] Gebreeyessus G.D. (2019). Status of hybrid membrane-ion-exchange systems for desalination: A comprehensive review. Appl. Water. Sci..

[5] Abdelfattah A., Hossain M.I., Cheng L. (2020). High-strength wastewater treatment using microbial biofilm reactor: A critical review. World J. Microb. Biot..

[6] Zhang C., Wu D., Ren H. (2020). Bioremediation of oil contaminated soil using agricultural wastes via microbial consortium. Sci. Rep..

[7] Dixit R.W., Malaviya D., Pandiyan K., Singh U.B., Sahu A., Shukia R., Singh B.P., Rai J.P., Sharma P.K., Lade H. (2015). Bioremediation of heavy metals from soil and aquatic environment: An overview of principles and criteria of fundamental processes. Sustainability.

[8] Mishra G.K. (2017). Microbes in heavy metal bioremediation: A review on current trends and patents. Recent Pat. Biotechnol..

[9] Diep P., Mahadevan R., Yakunin A.F. (2018). Heavy metal removal by bioaccumulation using genetically engineered microorganisms. Front. Bioeng. Biotechnol..

[10] Igiri B.E., Okoduwa S.I.R., Idoko G.O., Akabuogu E.P., Adeyi A.O., Ejiogu I.K. (2018). Toxicity and bioremediation of heavy metals contaminated ecosystem from Tannery wastewater: A review. J. Toxicol..

[11] Singh S., Kang S.H., Mulchandani A., Chen W. (2008). Bioremediation: Environmental clean-up through pathway engineering. Curr. Opin. Biotechnol..

[12] Kiadehi M.S.H., Amoozegar M.A., Asad S., Siroosi M. (2018). Exploring the potential of halophilic archaea for the decolorization of azo dyes. Water Sci. Technol..

[13] Azubuike C.C., Chikere C.B., Okpokwasili G.C. (2016). Bioremediation techniques-classification based on site of application: Principles, advantages, limitations and prospects. World J. Microbiol. Biotechnol..

[14] Waigi M.G., Sun K., Gao Y.Z. (2017). Sphingomonads in microbe-assisted phytoremediation: Tackling soil pollution. Trends Biotechnol..

[15] Tkacv R., Matrosova V.Y., Grichenko O.E., Gostinčar C., Volpe R.P., Klimenkova P., Gaidamakova E.K., Zhou C.E., Stewart B.J., Lyman M.G. (2018). Prospects for fungal bioremediation of acidic radioactive waste sites: Characterization and genome sequence of *Rhodotorula taiwanensis* MD1149. Front. Microbiol..

[16] Ali N., Dashti N., Khanafer M., Al-Awadhi H., Radwan S. (2020). Bioremediation of soils saturated with spilled crude oil. Sci. Rep..

[17] Rambabu K., Banat F., Pham Q.M., Ho S.-H., Ren N.-Q., Show P.L. (2020). Biological remediation of acid mine drainage: Review of past trends and current outlook. Environ. Sci. Technol..

[18] Cleary A., Lloyd J.R., Newsome L., Shaw S., Boothman C., Boshoff G., Atherton N., Morris K. (2019). Bioremediation of strontium and technetium contaminated groundwater using glycerol phosphate. Chem. Geol..

[19] Siliakus M.F., Van der Oost J., Kengen S.W.M. (2017). Adaptations of archaeal and bacterial membranes to variations in temperature, pH and pressure. Extremophiles.

[20] Rastädter K., Wurm D.J., Spadiut O., Quehenberger J. (2020). The cell membrane of *Sulfolobus* spp.–homeoviscous adaptation and biotechnological applications. Int. J. Mol. Sci..

[21] Vergara E., Neira G., González C., Cortez D., Dopson M., Holmes D.S. (2019). Evolution of predicted acid resistance mechanisms in the extremely acidophilic *Leptospirillum* genus. Genes.

[22] Guan N., Liu L. (2020). Microbial response to acid stress: Mechanisms and applications. Appl. Microbiol. Biotechnol..

[23] Salvador-Castell M., Tourte M., Ogar P.M. (2019). In search for the membrane regulators of archaea. Int. J. Mol. Sci..

[24] Golyshina O.V., Tran H., Reva O.N., Lemak S., Yakunin A.F., Goesmann A., Nechitaylo T.Y., LaCono V., Smedile F., Slesarev A. (2017). Metabolic and evolutionary patterns in the extreme acidophilic archaeon *Ferroplasma acidiphilum* Y^T^. Sci. Rep..

[25] Baker-Austin C., Dopson M. (2007). Life in acid: pH homeostasis in acidophiles. Trends Microbiol..

[26] Aono R., Ito M., Machida T. (1999). Contribution of the cell wall component teichuronopeptide to pH homeostasis and alkaliphily in the alkaliphile *Bacillus lentus* C-125. J. Bacteriol..

[27] Aono R. (1995). Assignment of facultatively alkaliphilic *Bacillus* sp. strain C-125 to *Bacillus lentus* group 3. Int. J. Syst. Bacteriol..

[28] Calamita H.G., Ehringer W.D., Koch A.L., Doyle R.J. (2001). Evidence that the cell wall of *Bacillus subtilis* is protonated during respiration. Proc. Natl. Acad. Sci. USA.

[29] Padan E., Bibi E., Ito M., Krulwich T.A. (2005). Alkaline pH homeostasis in bacteria: New insights. Biochim. Biophys. Acta Biomembr..

[30] Kitada M., Kosono S., Kudo T. (2000). The Na^+^/H^+^ antiporter of alkaliphilic *Bacillus* sp. Extremophiles.

[31] Fang H., Qin X.-Y., Zhang K.-D., Nie Y., Wu X.-L. (2018). Role of the Group 2 Mrp sodium/proton antiporter in rapid response to high alkaline shock in the alkaline- and salt-tolerant *Dietzia* sp. DQ12-45-1b. Appl. Microbiol. Biotechnol..

[32] Matsuno T., Goto T., Ogami S., Morimoto H., Yamazaki K., Inoue N., Matsuyama H., Yoshimune K., Yumoto I. (2018). Formation of proton motive force under low-aeration alkaline conditions in alkaliphilic bacteria. Front. Microbiol..

[33] Stancik L.M., Stancik D.M., Schmidt B., Barnhart D.M., Yoncheva Y.N., Slonczewski J.L. (2002). pH-Dependent expression of periplasmic proteins and amino acid catabolism in *Escherichia coli*. J. Bacteriol..

[34] Slonczewski J.L., Fujisawa M., Dopson M., Krulwich T.A. (2009). Cytoplasmic pH measurement and homeostasis in bacteria and archaea. Adv. Microb. Physiol..

[35] Wernick D.G., Pontrelli S.P., Pollock A.W., Liao J.C. (2016). Sustainable biorefining in wastewater by engineered extreme alkaliphile *Bacillus marmarensis*. Sci. Rep..

[36] Setati M.E. (2010). Diversity and industrial potential of hydrolase-producing halophilic/halotolerant eubacteria. Afr. J. Biotechnol..

[37] Gunde-Cimerman N., Plemenitaš A., Oren A. (2018). Strategies of adaptation of microorganisms of the three domains of life to high salt concentrations. FEMS Microbiol. Rev..

[38] Starhl H., Greie J.-C. (2008). The extremely halophilic archaeon *Halobacterium salinarum* R1 responds to potassium limitation by expression of the K^+^-transporting KdpFABC P-type ATPase and by a decrease in intracellular K^+^. Extremophiles.

[39] Engel M.B., Catchpole H.R. (2005). A microprobe analysis of inorganic elements in *Halobacterium salinarum*. Cell Biol. Int..

[40] Coker J.A., DasSarma P., Kumar J., Müller J.A., DasSarma S. (2007). Transcriptional profiling of model Archeon *Halobacterium* sp. NRC-1: Responses to changes in salinity and temperature. Saline Syst..

[41] Corral P., Amoozegar M.A., Ventosa A. (2020). Halophiles and their biomolecules: Recent advances and future applications in biomedicine. Mar. Drugs.

[42] Reed C.J., Bushnell S., Evilia C. (2014). Circular dichroism and fluorescence spectroscopy of cysteine-tRNA synthetase from *Halobacterium salinarum* ssp. NRC-1 demonstrates that group I cations are particularly effective in providing structure and stability to this halophilic protein. PLoS ONE.

[43] Brininger C., Spradlin S., Cobani L., Evilia C. (2018). The more adaptive to change, the more likely you are to survive: Protein adaptation in extremophiles. Semin. Cell Dev. Biol..

[44] Elcock A.H., McCammon J.A. (1998). Electrostatic contributions to the stability of halophilic proteins. J. Mol. Biol..

[45] León M.J., Hoffmann T., Sánchez-Porro C., Heider J., Ventosa A., Bremer E. (2018). Compatible solute synthesis and imported by the moderate halophile *Spiribacter salinus*: Physiology and genomics. Front. Microbiol..

[46] Le Borgne S., Paniagua D., Vazques-Duhalt R. (2008). Biodegradation of organic pollutants by halophilic bacteria and archaea. J. Mol. Microbiol. Biotechnol..

[47] Vandrich J., Pfeiffer F., Alfaro-Espinoza G., Kunte H.J. (2020). Contribution of mechanosensitive channels to osmoadaptation and ectoine excretion in *Halomonas elongata*. Extremophile.

[48] Collins T., Margesin R. (2019). Psychrophilic lifestyles: Mechanisms of adaptation and biotechnological tools. Appl. Microbiol. Biotechnol..

[49] De Maayer P., Anderson D., Cary C., Cowan D.A. (2014). Some like it cold: Understanding the survival strategies of psychrophiles. EMBO Rep..

[50] Yoshimune K., Galkin A., Kulakova L., Yoshimura T., Esaki N. (2005). Cold-active DnaK of an Antarctic psychrotroph *Shewanella* sp. Ac10 supporting the growth of dnaK-null mutant of *Escherichia coli* at cold temperatures. Extremophiles.

[51] Białkowska A., Majewska E., Olczak A., Twarda-Clapa A. (2020). Ice binding proteins: Diverse biological roles and applications in different types of industry. Biomolecules.

[52] Ranawat P., Rawat S. (2017). Stress response physiology of thermophiles. Arch. Microbiol..

[53] Sprott G.D., Meloche M., Richards J.C. (1991). Proportions of diether, macrocyclic diether, and tetraether lipids in *Methanococcus jannaschii* grown at different temperatures. J. Bacteriol..

[54] Mansilla M.C., Cybulski L.E., Albanesi D., de Mendoza D. (2004). Control of membrane lipid fluidity by molecular thermosensors. J. Bacteriol..

[55] Valenti A., Perugino G., Rossi M., Ciaramella M. (2011). Positive supercoiling in thermophiles and mesophiles: Of the good and evil. Biochem. Soc. Trans..

[56] Sieck G. (2015). Life at the extreme: Physiological adaptation. Physiology.

[57] Wang Q., Cen Z., Zhao J. (2015). The survival mechanisms of thermophiles at high temperatures: An angle of omics. Physiology.

[58] Ferreira A.C., Nobre M.F., Rainey F.A., Silva M.T., Wait R., Burghardt J., Chung A.P., Da Costa M.S. (1997). *Deinococcus geothermalis* sp. nov. and *Deinococcus murrayi* sp. nov., two extremely radiation-resistant and slightly thermophilic species from hot springs. Int. J. Syst. Evol..

[59] Liu Z., Kim M.C., Wang L., Zhu G., Zhang Y., Huang Y., Wei Z., Danzeng W., Peng F. (2017). *Deinococcus taklimakanensis* sp. nov. isolated from desert soil. Int. J. Syst. Evol..

[60] Srinivasan S., Lim S.Y., Lim J.-H., Jung H.-Y., Kim M.K. (2017). *Deinococcus rubrus* sp. nov., a bacterium isolated from Antarctic coastal sea water. J. Microbiol. Biotechnol..

[61] Park M.R., Song J.H., Nam G.G., Joung Y.C., Zhao L., Kim M.-K., Cho J.C. (2018). *Deinococcus lacus* sp. nov., a gamma radiation-resistant bacterium isolated from an artificial freshwater pond. Int. J. Syst. Evol..

[62] Tanner K., Molina-Menor E., Latorre-Pérez A., Vidal-Verdú À., Vilanova C., Peretó J., Porcar M. (2020). Extremophilic microbial communities on photovoltaic panel surfaces: A two-year study. Microb. Biotechnol..

[63] Slade D., Radman M. (2011). Oxidative stress resistance in *Deinococcus radiodurans*. Microbiol. Mol. Biol. Rev..

[64] Radjpurohit Y.S., Bihani S.C., Waldor M.K., Misra H.S. (2016). Phosphorylation of *Deinococcus radiodurans* RecA regulates its activity and may contribute to radioresistance. J. Biol. Chem..

[65] Tanaka M., Earl A.M., Howell H.A., Park M.J., Eisen J.A., Peterson S.N., Battista J.R. (2004). Analysis of *Deinococcus radiodurans*’s transcriptional response to ionizing radiation and desiccation reveals novel proteins that contribute to extreme radioresistance. Genetics.

[66] Lim S.Y., Jung J.H., Blanchard L., de Groot A. (2019). Conservative and diversity of radiation and oxidative stress resistance mechanisms in *Deinococcus radiodurans*. FEMS Microbiol. Rev..

[67] Jin M., Xiao A., Zhang Z., Huang H., Jiang L. (2019). The diversity and commonalities of the radiation-resistance mechanisms of *Deinococcus* and its up-to-date applications. AMB Express..

[68] Floc’h K., Lacroix F., Servant P., Wong Y.-S., Kelman J.-P., Bourgeois D., Timmins J. (2019). Cell morphology and nucleoid dynamic in dividing *Deinococcus radiodurans*. Nat. Commun..

[69] Jeong S.-W., Jung J.H., Kim M.K., Seo H.S., Lim H.-M., Lim S.Y. (2016). The three catalases in *Deinococcus radiodurans*: Only two show catalase activity. Biochem. Biophys. Res. Commun..

[70] Maqbool I., Sudharsan M., Kanimozhi G., Alrashood S.T., Khan H.A., Prasad N.R. (2020). Crude cell-free extract from *Deinococcus radiodurans* exhibit anticancer activity by inducing apoptosis in triple-negative breast cancer cells. Front. Cell Dev. Biol..

[71] Choi J.Y., Lee K.J., Lee P.C. (2019). Characterization of carotenoid biosynthesis in newly isolated *Deinococcus* sp. AJ005 and investigation of the effects of environmental conditions on cell growth and carotenoid biosynthesis. Mar. Drugs.

[72] Daly M.J., Gaidamakova E.K., Matrosova V.Y., Kiang J.G., Fukumoto R., Lee D.-Y., Wehr N.B., Viteri G.A., Berlett B.S., Levine R.L. (2010). Small-molecule antioxidant proteome-shields in *Deinococcus radiodurans*. PLoS ONE.

[73] Santos S.P., Yang Y., Rosa M.T.G., Rodrigues M.A.A., De La Tour C.B., Sommer S., Teixeira M., Carrondo M.A., Cloetens P., Abreu I.A. (2019). The interplay between Mn and Fe in *Deinococcus radiodurans* triggers cellular protection during paraquat-induced oxidative stress. Sci. Rep..

[74] Robinson C.K., Webb K., Kaur A., Jaruga P., Dizdaroglu M., Baliga N.S., Place A., DiRuggiero J. (2011). A major role for nonenzymatic antioxidant processes in the radioresistance of *Halobacterium salinarum*. J. Bacteriol..

[75] Gumulya Y., Boxall N.J., Khaleque H.N., Santala V., Carlson R.P., Kaksonen A.H. (2018). In a quest for engineering acidophiles for biomining applications: Challenges and opportunities. Genes.

[76] Navarro C.A., Von Bernath D., Jerez C.A. (2013). Heavy metal resistance strategies of acidophilic bacteria and their acquisition: Importance for biomining and bioremediation. Biol. Res..

[77] Saavedra A., Aguirre P., Gentina J.C. (2020). Biooxidation of iron by *Acidithiobacillus ferroxidans* in the presence of D-galactose: Understanding its influence on the production of EPS and cell tolerance to high concentration of iron. Front. Microbiol..

[78] Jafari M., Abdollahi H., Shafaei S.Z., Gharabaghi M., Jafari H., Akcil A. (2019). Acidophilic bioleaching: A review on the process and effect of organic-inorganic reagents and materials on its efficiency. Min. Proc. Ext. Met. Rev..

[79] Brierley J.A. (2008). A perspective on developments in biohydrometallurgy. Hydrometallurgy.

[80] Chen P., Yan L., Leng F., Nan W., Yue X., Zheng Y., Feng N., Li H. (2011). Bioleaching of realgar by *Acidithiobacillus ferrooxidans* using ferrous iron and elemental sulfur as the sole and mixed energy sources. Bioresour. Technol..

[81] Zhang S., Yan L., Xing W., Chen P., Zhang Y., Wang W. (2018). *Acidothiobacillus ferrooxidans* and its potential application. Extremophiles.

[82] Romero-González M., Nwaobi B.C., Hufton J.M., Gilmour D.J. (2016). Ex-situ bioremediation of U(VI) from contaminated mine water using *Acidothiobacillus ferrooxidans* strains. Front. Environ. Sci..

[83] Jameson E., Rowe O.F., Hallberg K.B., Johnson D.B. (2010). Sulfidogenesis and selective precipitation of metals at low pH mediated by *Acidithiobacillus* spp. and acidophilic sulfate-reducing bacteria. Hydrometallurgy.

[84] Okibe N., Maki M., Nakayama D., Sasaki K. (2016). Microbial recovery of vanadium by the acidophilic bacterium, *Acidocella aromatica*. Biotechnol. Lett..

[85] Chakravarty R., Banerjee P.C. (2012). Mechanism of cadmium binding on the cell wall of an acidophilic bacterium. Bioresour. Technol..

[86] Beolchini F., Dell’Anno A., De Propris L., Ubaldini S., Cerrone F., Danovaro R. (2009). Auto- and heterotrophic acidophilic bacteria enhance the bioremediation efficiency of sediments contaminated by heavy metals. Chemosphere.

[87] Gupta A., Sar P. (2020). Characterization and application of an anaerobic, iron and sulfate reducing bacterial culture in enhanced bioremediation of acid mine drainage impacted soil. J. Environ. Sci. Health C.

[88] Abd-Elnaby H., Abou-Elela G.M., El-Sersy N.A. (2011). Cadmium resisting bacteria in Alexandria Eastern Harbor (Egypt) and optimization of cadmium bioaccumulation by *Vibrio harveyi*. Afr. J. Biotechnol..

[89] Iyer A., Mody K., Jha B. (2005). Biosorption of heavy metals by a marine bacterium. Mar. Pollut. Bull..

[90] Ӧzdemir S., Kilinc E., Poli A., Nicolaus B. (2013). Biosorption of heavy metals (Cd^2+^, Cu^2+^, Co^2+^, and Mn^2+^) by thermophilic bacteria, *Geobacillus thermantarcticus* and *Anoxybacillus amylolyticus*: Equilibrium and kinetic studies. Bioremediat. J..

[91] Eslami N., Kermanshahi R.K., Erfan M. (2013). Studying the stability of S-layer protein of *Lactobacillus acdiophilius* ATCC 4356 in simulated gastrointestinal fluids using SDS-PAGE and circular dichroism. Iran J. Pharm. Res..

[92] Liu S., Zheng Y., Ma Y., Sarwar A., Zhao X., Luo T., Yang Z. (2019). Evaluation and proteomic analysis of lead adsorption by lactic acid bacteria. Int. J. Mol. Sci..

[93] Gerbino E., Mobili P., Tymczyszyn E., Fausto R., Gómez-Zavaglia A. (2011). FTIR spectroscopy structural analysis of the interaction between *Lactobacillus kefir* S-layers and metal ions. J. Mol. Struct..

[94] Kashefi K., Lovely D.R. (2000). Reduction of Fe(III), Mn(IV), and toxic metals at 100 °C by *Pyrobacculum islandicum*. Appl. Environ. Microbiol..

[95] Feitkenhauer H., Muller R., Markl H. f. (2003). Degradation of polycyclic aromatic hydrocarbons and long chain alkanes at 60-70 degrees C by *Thermus * and *Bacillus* spp.. Biodegradation.

[96] Nazina T.N., Tourova T.P., Poltaraus A.B., Novikova E.V., Grigoryan A.A., Ivanova A.E., Lysenko A.M., Petrunyaka V.V., Osipov G.A., Belyaev S.S. (2001). Taxonomic study of aerobic thermophilic bacilli: Descriptions of Geobacillus subterraneus gen. nov., sp. nov., and Geobacillus uzenensis sp. nov. from petroleum reservoirs and transfer of Bacillus stearothermophilus, Bacillus thermocatenulatus, Bacillus thermoleovorans, Bacillus kaustophilus, Bacillus thermodenitrificans to Geobacillus as the new combinations G. stearothermophilus, G. th. Int. J. Syst. Evol. Microbiol..

[97] Feng L., Wang W., Cheng J., Ren Y., Zhao G., Gao C., Tang Y., Liu X., Han W., Peng X. (2007). Genome and proteome of long-chain alkane degrading *Geobacillus thermodenitrificans* NG80-2 isolated from a deep-subsurface oil reservoir. Proc. Natl. Acad. Sci. USA.

[98] Sood N., Lal B. (2008). (2008). Isolation and characterization of a potential paraffin-wax degrading thermophilic bacterial strain *Geobacillus kaustophilus* TERI NSM for application in oil wells with paraffin deposition problems. Chemosphere.

[99] Sun Y., Ning Z., Yang F., Li X. (2015). Characteristics of newly isolated *Geobacillus* sp. ZY-10 degrading hydrocarbons in crude oil. Pol. J. Microbiol..

[100] Zhang J., Zhang X., Liu J., Li R., Shen B. (2012). Isolation of a thermophilic bacterium, *Geobacillus* sp. SH-1, capable of degrading aliphatic hydrocarbons and naphthalene simultaneously, and identification of its naphthalene degrading pathway. Bioresour. Technol..

[101] Elumalai P., Parthipan P., Karthikeyan O.P., Rajasekar A. (2017). Enzyme-mediated biodegradation of long-chain n-alkanes (C_32_ and C_40_) by thermophilic bacteria. 3 Biotech..

[102] Lin X., Yang B., Shen J., Du N. (2009). Biodegradation of crude oil by an Arctic psychrotrophic bacterium *Pseudoalteromomas* sp. P29. Curr. Microbiol..

[103] Gentile G., Bonsignore M., Santisi S., Catalfamo M., Giuliano L., Genovese L., Yakimov M.M., Denaro R., Genovese M., Cappello S. (2016). Biodegradation potentiality of psychrophilic bacterial strain *Oleispira antarctica* RB-8(T). Mar. Pollut. Bull..

[104] Gao W., Cui Z., Li Q., Xu G., Jia X., Zheng L. (2013). *Marinobacter nanhaiticus* sp. nov., polycyclic aromatic hydrocarbon-degrading bacterium isolated from the sediment of the South China Sea. Antonie Van Leeuwenhoek.

[105] Al-Mailem D.M., Eliyas M., Radwan S.S. (2013). Oil-bioremediation potential of two hydrocarbonoclastic, diazotrophic *Marinobacter* strains from hypersaline areas along the Arabian Gulf coasts. Extremophiles.

[106] Ibrahim I.M., Konova S.V., Sigida E.N., Lyubun E.V., Muratova A.Y., Fedonenko Y.P., Elbanna K. (2020). Bioremediation potential of a halophilic *Halobacillus* sp. strain EG1HP4QL: Exopolysaccharide production, crude oil degradation, and heavy metal tolerance. Extremophile.

[107] Gutierrez T., Berry D., Yang T., Mishamandani S., McKay L., Teske A., Aitken M.D. (2013). Role of exopolysaccharide (EPS) in the fate of the oil released during the Deepwater Horizon oil spill. PLoS ONE.

[108] Sadegh H., Ali G.A.M., Gupta V.K., Makhlouf A.S.H.M., Shahryari-ghoshekandi R., Nadagouda M.N., Sillanpää M., Megiel E. (2017). The role of nanomaterials as effective adsorbents and their applications in wastewater treatment. J. Nanostruct. Chem..

[109] Kim Y.-H., Jeon J.H., Hong S.H., Rhim W.-K., Lee Y.-S., Youn H.W., Chung J.-K., Lee M.C., Lee D.S., Kang K.W. (2011). Tumor targeting and imaging using cyclic RGD-PEGlated gold nanoparticle probes with directly conjugated iodine-125. Small.

[110] Choi M.H., Shim H.E., Yun S.J., Park S.H., Choi D.S., Jang B.-S., Choi Y.J., Jeon J.H. (2016). Gold-nanoparticle-immobilized desalting column for highly efficient and specific removal of radioactive iodine in aqueous media. Acs Appl. Mater. Interspace.

[111] Mushtaq S., Yun S.-J., Yang J.E., Jeong S.-W., Shim H.E., Choi M.H., Park S.H., Choi Y.J., Jeon J.H. (2017). Efficient and selective removal of radioactive iodine anions using engineered nanocomposite membranes. Environ. Sci. Nano.

[112] Lloyd J.R. (2003). Microbial reduction of metals and radionuclides. FEMS Microbiol. Rev..

[113] Kumar R., Singh S., Singh O.V. (2007). Bioremediation of radionuclides: Emerging technologies. OMICS.

[114] Prakash D., Gabani P., Chandel A.K., Ronen Z., Singh O.V. (2013). Bioremediation: A genuine technology to remediate radionuclides from the environment. Microb. Biotechnol..

[115] Shukla A., Parmar P., Sarar M. (2017). Radiation, radionuclides and bacteria: An in-perspective review. J. Environ. Radioact..

[116] Wildung R.E., Gorby Y.A., Krupka K.M., Hess N.J., Li S.W., Plymale A.E., McKinley J.P., Fredrickson J.K. (2000). Effect of electron donor and solution chemistry on products of dissimilatory reduction of technetium by *Shewanella putrefaciens*. Appl. Environ. Microbiol..

[117] Istok J.D., Senko J.M., Krumholz L.R., Watson D., Bogle M.A., Peacock A., Chang Y.-J., White D.C. (2004). In situ bioreduction of technetium and uranium in a nitrate-contaminated aquifer. Environ. Sci. Technol..

[118] Panak P.J., Nitsche H. (2001). Interaction of aerobic soil bacteria with plutonium(VI). Radiochim. Acta..

[119] Kim S.-J., Koh D.-C., Park S.-J., Cha I.-T., Park J.-W., Na J.-H., Roh Y., Ko K.-S., Kim K.J., Rhee S.-K. (2012). Molecular analysis of spatial variation of iron-reducing bacteria in riverine alluvial aquifers of the Mankyeong River. J. Microbiol..

[120] Anderson R.T., Vrionis H.A., Ortiz-Bernard I., Resch C.T., Long P.E., Dayvault R., Karp K., Metzler D.R., Peacock A., White D.C. (2003). Stimulating the in situ activity of *Geobacter* species to remove uranium from the groundwater of a uranium-contaminated aquifer. Appl. Environ. Microbiol..

[121] Ferreira A.C., Nobre M.F., Moore E., Rainey F.A., Battista J.R., Da Costa M.S. (1999). Characterization and radiation resistance of new isolates of *Rubrobacter radiotolerans* and *Rubrobacter xylanophilus*. Extremophiles.

[122] Billi D., Friedmann E.I., Hofer K.G., Caiola M.G., Ocampo-Friedmann R. (2000). Ionizing-radiation resistance in the desiccation-tolerant cyanobacterium *Chroococcidiopsis*. Appl. Environ. Microbiol..

[123] Brim H., Mcfarlan S.C., Fredrickson J.K., Minton K.W., Zhai M., Wackett L.P., Daly M.J. (2000). Engineering *Deinococcus radiodurans* for metal remediation in radioactive mixed waste environments. Nat. Biotechnol..

[124] Daly M.J. (2000). Engineering radiation-resistant bacteria for environmental biotechnology. Curr. Opin. Biotechnol..

[125] Jeong S.-W., Choi Y.J. (2017). Research perspective of an extremophilic bacterium, *Deinococcus radiodurans* on bioremediation of radioactive wastes. Appl. Chem. Eng..

[126] Fredrickson J.K., Kostandarithes H.M., Li S.W., Plymale A.E., Daly M.J. (2000). Reduction of Fe(III), Cr(VI), U(VI), and Tc(VII) by *Deinococcus radiodurans* R1. Appl. Environ. Microbiol..

[127] Appukuttan D., Rao A.S., Apte S.K. (2006). Engineering of *Deinococcus radiodurans* R1 for bioprecipitation of uranium from dilute nuclear waste. Appl. Environ. Microbiol..

[128] Misra C.S., Appukuttan D., Kantamreddi V.S.S., Rao A.S., Apte S.K. (2012). Recombinant, *D. radiodurans* cells for bioremediation of heavy metals from acidic/neutral aqueous wastes. Bioeng. Bugs.

[129] Appukuttan D., Seetharam C., Padma N., Rao A.S., Apte S.K. (2011). PhoN-expressing, lyophilized, recombinant *Deinococcus radiodurans* cells for uranium bioprecipitation. J. Biotechnol..

[130] Gogada R., Singh S.S., Lunavat S.K., Pamarthi M.M., Rodrigue A., Vadivelu B., Phanithi P.-B., Gopala V., Apte S.K. (2015). Engineered *Deinococcus radiodurans* R1 with NiCoT genes for bioremoval of trace cobalt from spent decontamination solutions of nuclear power reactors. Appl. Microbiol. Biotechnol..

[131] Choi M.H., Jeong S.-W., Shim H.E., Yun S.J., Mushtaq S., Choi D.S., Jang B.-S., Jung E.Y., Choi Y.J., Jeon J.H. (2017). Efficient bioremediation of radioactive iodine using biogenic gold nanomaterial-containing radiation-resistant bacterium, *Deinococcus radiodurans* R1. Chem. Comm..

[132] Kulkarni R.R., Shaiwale N.S., Deobagkar D.N., Deobagkar D.D. (2015). Synthesis and extracellular accumulation of silver nanoparticles by employing radiation-resistant *Deinococcus radiodurans*, their characterization, and determination of bioactivity. Int. J. Nanomed..

[133] Beeler E., Singh O.V. (2016). Extremophiles as sources of inorganic bio-nanoparticles. World, J. Microbiol. Biotechnol..

[134] Li J., Li Q., Ma X., Tian B., Yu J., Dai S., Weng Y., Hua Y. (2016). Biosynthesis of gold nanoparticles by the extreme bacterium *Deinococcus radiodurans* and an evaluation of their antibacterial properties. Int. J. Nanomed..

[135] Chen A., Contreras L.M., Keitz B.K. (2017). Imposed environmental stresses facilitate cell-free nanoparticle formation by *Deinococcus radiodurans*. Appl. Environ. Microbiol..

[136] Kitjanukit S., Sasaki K., Okibe N. (2019). Production of highly catalytic, archaeal Pd(0) bionanoparticles using *Sulfolobus tokodaii*. Extremophiles.

[137] Okibe N., Nakayama D., Matsumoto T. (2017). Palladium bionanoparticles production from acidic Pd(II) solutions and spent catalyst leachate using acidophilic Fe(II)-reducing bacteria. Extremophiles.

[138] Crini G., Lichtfouse E. (2019). Advantages and disadvantages of techniques used for wastewater treatment. Environ. Chem. Lett..

[139] Ren J., Lee J.G., Na D.K. (2020). Recent advances in genetic engineering tools based on synthetic biology. J. Microbiol..

[140] Wang Y., Wang D., Wang X., Tao H., Feng E., Zhu L., Pan C., Wang B., Liu C., Liu X. (2019). Highly efficient genome engineering in *Bacillus anthracis* and *Bacillus cereus* using CRISPR/Cas9 system. Front. Microbiol..

[141] Marques C.R. (2018). Extremophilic microfactories: Application in metal and radionuclide bioremediation. Front. Microbiol..

[142] Giovanella P., Vieira G.A.L., Ramos Otero I.V., Pellizzer E.P., de Jesus Fontes B., Sette L.D. (2020). Metal and organic pollutants bioremediation by extremophile microorganisms. J. Hazard. Mater..

